# Artificial Intelligence in Diabetic Retinopathy and Diabetic Macular Edema: A Narrative Review

**DOI:** 10.3390/bioengineering12121342

**Published:** 2025-12-09

**Authors:** Anđela Jukić, Josip Pavan, Miro Kalauz, Andrijana Kopić, Vedran Markušić, Tomislav Jukić

**Affiliations:** 1 Department of Ophthalmology, University Hospital Dubrava, 10000 Zagreb, Croatia; 2 Department of Ophthalmology, University Hospital Centre Zagreb, 10000 Zagreb, Croatia; 3 School of Medicine, University of Zagreb, 10000 Zagreb, Croatia; 4 Department of Ophthalmology, University Hospital Centre Osijek, 31000 Osijek, Croatia; 5 Faculty of Medicine Osijek, Josip Juraj Strossmayer University of Osijek, 31000 Osijek, Croatia

**Keywords:** diabetic retinopathy, diabetic macular edema, artificial intelligence, deep learning, screening, retinal imaging, OCT

## Abstract

Diabetic retinopathy (DR) and diabetic macular edema (DME) remain major causes of vision loss among working-age adults. Artificial intelligence (AI), particularly deep learning, has gained attention in ophthalmic imaging, offering opportunities to improve both diagnostic accuracy and efficiency. This review examined applications of AI in DR and DME published between 2010 and 2025. A narrative search of PubMed and Google Scholar identified English-language, peer-reviewed studies, with additional screening of reference lists. Eligible articles evaluated AI algorithms for detection, classification, prognosis, or treatment monitoring, with study selection guided by PRISMA 2020. Of 300 records screened, 60 met the inclusion criteria. Most reported strong diagnostic performance, with sensitivities up to 96% and specificities up to 98% for detecting referable DR on fundus photographs. Algorithms trained on optical coherence tomography (OCT) data showed high accuracy for identifying DME, with area under the receiver operating characteristic curve (AUC) values frequently exceeding 0.90. Several models also predicted anti-vascular endothelial growth factor (anti-VEGF) treatment response and recurrence of fluid with encouraging results. Autonomous AI tools have gained regulatory approval and have been implemented in clinical practice, though performance can vary depending on image quality, device differences, and patient populations. Overall, AI demonstrates strong potential to improve screening, diagnostic consistency, and personalized care, but broader validation and system-level integration remain necessary.

## 1. Introduction

Diabetic retinopathy (DR) and diabetic macular edema (DME) remain among the leading causes of vision impairment in working-age adults worldwide. The global burden of diabetes is rising rapidly, with projections suggesting that nearly 700 million individuals will be affected by 2045, leading to an estimated 160 million cases of DR and over 28 million cases of DME within the same timeframe [1].

DR represents a microvascular complication of diabetes characterized by progressive damage to the retinal capillaries, resulting in ischemia, microaneurysms, hemorrhages, and neovascularization. DME, a vision-threatening manifestation of DR, arises from the breakdown of the blood-retinal barrier and the accumulation of extracellular fluid within the macula. While DR reflects the widespread microvascular injury caused by chronic hyperglycemia, DME specifically develops when vascular leakage and inflammatory processes increase retinal permeability near the fovea. Risk factors such as long disease duration, poor glycemic control, hypertension, and renal impairment further elevate the likelihood of DME development.

Vision loss from DR is largely preventable with early detection and timely intervention, yet access to consistent screening remains a major challenge. In many regions, fewer than one-third of individuals with diabetes undergo annual dilated retinal examinations, despite established clinical guidelines recommending regular screening [2,3,4].

DME, the primary cause of visual impairment in DR, may occur at any stage of retinopathy and often remains asymptomatic until central vision is affected [5]. The advent of optical coherence tomography (OCT) has revolutionized DME diagnosis and monitoring by providing high-resolution, cross-sectional visualization of retinal architecture. OCT reveals hallmark features such as retinal thickening, intraretinal cystoid spaces, subretinal fluid, and hard exudates, which are central to disease staging and therapeutic decision-making [5].

Over the past decade, artificial intelligence (AI), particularly deep learning, has emerged as a transformative technology in ophthalmology. AI systems employing convolutional neural networks (CNNs) can interpret complex imaging patterns in fundus photographs and OCT scans with speed and consistency comparable to expert performance [6,7,8]. Early efforts in DR screening were driven by the availability of large retinal image datasets and the public health importance of the disease, facilitating the development and regulatory approval of autonomous AI-based screening systems. Since then, AI applications have broadened to include disease severity grading, risk prediction, and assessment of treatment response, especially in the context of anti-vascular endothelial growth factor (anti-VEGF) therapy for DME.

Modern AI models now support diverse clinical tasks, including lesion detection (e.g., microaneurysms, hemorrhages, retinal fluid), disease staging, progression prediction, and personalized treatment planning [9]. These tools have the potential to enhance diagnostic precision, reduce clinician workload, and improve patient outcomes.

While the primary focus of this review is on AI applications in DR and DME, occasional references to related retinal diseases are included only to illustrate the translational relevance and cross-applicability of emerging AI frameworks in ophthalmology.

This review explores the integration of AI in the detection, classification, and management of DR and DME, with a particular emphasis on OCT-based imaging. We summarize the clinical utility of AI in screening and grading, highlight currently available tools (including FDA cleared systems), and discuss how AI driven decision support is being incorporated into real-world retinal practice.

## 2. Methodology

**Figure 1:**
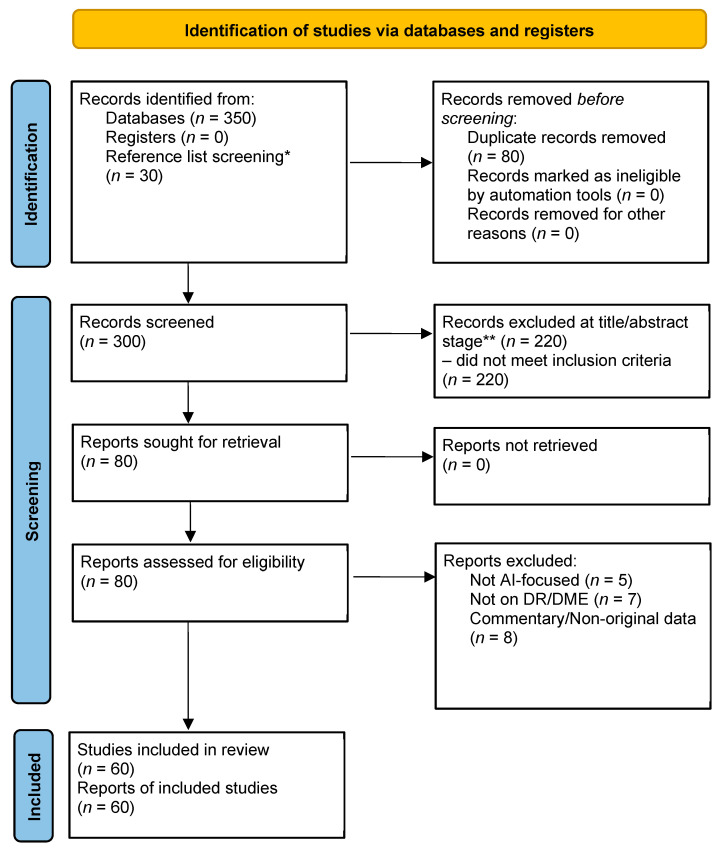
PRISMA 2020 flow diagram. * In total, 30 records were additionally identified through reference list screening; ** 220 records were excluded at title/abstract stage: did not meet inclusion criteria.

This article is a narrative review that synthesizes evidence on artificial intelligence (AI) in diabetic retinopathy (DR) and diabetic macular edema (DME), with selected elements of PRISMA 2020 used solely to improve transparency of the search and reporting process. Searches were conducted in PubMed and Google Scholar, covering publications from 1 January 2010 to 10 June 2025. Databases such as Embase, Web of Science, and IEEE Xplore were not systematically searched due to the narrative scope and resource limitations, which may have resulted in omission of some engineering-focused AI studies. The search strategy combined terms for diabetic retinopathy, diabetic macular edema, and artificial intelligence methods using Boolean operators (e.g., “diabetic retinopathy” OR “diabetic macular edema”) AND (“artificial intelligence” OR “deep learning” OR “machine learning”). Studies were eligible if they evaluated AI applied to retinal imaging for DR or DME detection, classification, prediction, or treatment monitoring. Studies were excluded if they did not involve AI, lacked imaging-based outcomes, or did not report quantitative performance metrics. Title/abstract and full-text screening as well as data extraction were performed by one reviewer (A.J.). No dual-reviewer agreement metrics were calculated. This introduces a possible risk of selection bias and is acknowledged as a limitation. The selection process is illustrated in the PRISMA 2020 flow diagram (Figure 1).

## 3. Results

### 3.1. AI for Detection and Diagnostic Accuracy in DR and DME

Over the past decade, artificial intelligence (AI) algorithms have progressed from experimental prototypes to clinically validated tools for diabetic retinopathy (DR) screening. These systems are typically trained on large datasets comprising hundreds of thousands of color fundus photographs, enabling the detection of referable DR, defined as moderate non-proliferative DR or worse and/or diabetic macular edema (DME), with high diagnostic accuracy.

Gulshan et al. reported that a deep convolutional neural network (CNN) achieved 90.3% sensitivity and 98.1% specificity for detecting referable DR from fundus images, a performance comparable to or exceeding that of expert ophthalmologists [9]. Building on this success, Abràmoff et al. evaluated an autonomous AI system in primary care settings, demonstrating 87.2% sensitivity and 90.7% specificity for more-than-mild DR, results that led to the first FDA approval of an autonomous AI diagnostic system in any medical field in 2018 [10]. Similarly, EyeArt^®^ (Eyenuk Inc., Woodland Hills, CA, USA) achieved 96% sensitivity and 88% specificity for referable DR in a multicenter clinical trial, earning FDA clearance shortly thereafter [11].

These autonomous systems analyze fundus images to identify characteristic lesions, such as microaneurysms, hemorrhages, and exudates, and determine referral need. By 2025, several such tools had been deployed in real-world settings, including pharmacy and community based screening stations, aiming to expand access to DR detection.

Although color fundus photography remains the standard for DR screening, optical coherence tomography (OCT) is indispensable for detecting and monitoring DME. AI models trained on OCT data can identify intraretinal cysts, retinal thickening, and subretinal fluid. Tang et al. trained a deep-learning system on 73,746 B-scans from three OCT platforms, achieving area-under-the-curve (AUC) values between 0.94 and 0.97 for detecting and classifying DME across devices [12]. However, large-scale screening using OCT remains impractical due to the high cost of devices, the technical complexity of image acquisition, and limited availability in community settings [13].

A recent meta-analysis found that combining OCT with fundus imaging does not significantly improve AI screening performance compared with fundus photographs alone [14]. Fundus based AI systems have demonstrated the ability to identify DME without reliance on OCT. For instance, Varadarajan et al. trained an algorithm on more than 6000 fundus photographs to predict OCT derived central retinal thickness, achieving 85% sensitivity and 80% specificity for detecting center-involved DME, performance that outperformed some retina specialists [15]. These findings support a hybrid model in which fundus based AI tools triage patients, with OCT reserved for confirmation and treatment planning.

Beyond single disease detection, AI has also demonstrated the capacity to identify multiple retinal pathologies from OCT scans. A collaboration between DeepMind (Google Health) and Moorfields Eye Hospital developed a two-stage deep-learning pipeline for three-dimensional OCT analysis. This model segmented pathologic features and provided referral recommendations for more than 50 retinal conditions, including DME and proliferative DR. According to the results published in *Nature Medicine*, the model achieved over 94% accuracy in identifying cases requiring urgent referral and detected all sight-threatening diseases, providing interpretable visual explanations through OCT overlays [7,16].

Despite promising trial results, real world performance can vary. AI systems validated under controlled conditions often show reduced accuracy in routine practice. A large scale validation of Google’s DR algorithm in Thailand revealed a drop in specificity when deployed in primary care, largely due to image quality variation and workflow differences [13]. Reported real-world sensitivities for AI-based DR screening range from 51 to 86%, with specificities between 60 and 84% [13]. This variability raises concerns about false negatives, missed cases of disease, and false positives leading to unnecessary referrals, often resulting from limited diversity in training data, variable image quality, or overfitting to research datasets.

Addressing these challenges requires continuous performance monitoring, domain adaptation, and retraining across diverse populations. As Kelly et al. highlight, unresolved issues related to data quality, generalizability, regulation, and workflow integration remain barriers to clinical impact [17]. Keane and Topol call for rigorous validation and regulatory safeguards to promote safe AI adoption [18], while Ting et al. emphasize that ophthalmic AI must bridge the gap between technical performance and clinical practicality [19].

Even with these limitations, AI-based DR screening has demonstrated clear clinical value. In resource limited settings, Bellemo et al. showed that deep-learning AI achieved high accuracy in detecting referable and vision threatening DR alongside DME [20]. A multicenter, real world head-to-head evaluation of seven autonomous AI systems found that only one matched human graders in both sensitivity and specificity, underscoring the need for rigorous external validation before widespread adoption [21].

In the United States, the establishment of Current Procedural Terminology (CPT) code 92229 in 2021 created a reimbursement pathway for autonomous AI-based diabetic retinopathy (DR) screening, encouraging broader clinical adoption [22]. Nevertheless, adoption remains modest. A longitudinal analysis from 2019 to 2023 found that fewer than 5% of diabetic patients underwent AI-based screening [23]. Importantly, among those screened, ophthalmology referral and OCT confirmed disease detection rates were significantly higher, suggesting that AI is identifying otherwise unrecognized pathology [24].

Deep-learning algorithms consistently achieve high sensitivity and specificity for referable DR, often outperforming general ophthalmologists and approaching the accuracy of retina specialists [25]. One of AI’s key advantages is diagnostic consistency, free from fatigue and inter-observer variability, enabling reliable detection of subtle lesions across large datasets. For DME, AI applied to OCT demonstrates similarly strong performance in identifying hallmark features, though scalability remains limited. A practical approach is staged screening: fundus-based AI for population level detection, followed by OCT confirmation and management.

AI has the potential to expand DR and DME screening through both telemedicine and in clinic integration. Its long-term value will depend on robust validation across diverse populations, seamless workflow integration, and continuous monitoring to ensure that AI systems remain safe, accurate, and equitable.

### 3.2. AI in Disease Staging and Progression Monitoring

Accurate staging of diabetic retinopathy (DR) and diabetic macular edema (DME) is fundamental to appropriate treatment decisions. DR severity is typically classified using the Early Treatment Diabetic Retinopathy Study (ETDRS) scale, ranging from mild and moderate non-proliferative DR (NPDR) to proliferative DR (PDR), while DME is assessed by optical coherence tomography (OCT) and categorized as center involved or non-center involved, often based on central subfield thickness (CST).

Recent advances in deep learning (DL) have enabled automated staging of both DR and DME directly from imaging. Some AI models not only detect referable DR but also provide staging with accuracy of expert graders [26]. For instance, one study reported ~95% classification accuracy in categorizing OCT images according to retinal pathology severity using a DL framework [27]. These tools reduce the subjectivity inherent in human grading and allow for standardized triage in large screening programs. For example, moderate NPDR or any DME cases can be flagged for ophthalmologist referral, while mild NPDR might be deferred to routine surveillance.

AI is also changing how we predict disease progression, opening the door to personalized follow-up intervals and earlier preventive interventions. Several predictive models have been developed that incorporate serial imaging and systemic data to forecast DR worsening. Arcadu et al. utilized time series fundus photographs to predict ETDRS worsening within one year, achieving an area under the curve (AUC) of ~0.79 [28]. When systemic factors such as HbA1c, diabetes duration, and blood pressure were added, predictive performance improved further [29]. More recently, Rom et al. developed a DL model that predicted conversion to PDR up to 3–5 years in advance with an AUC of 0.82 [30].

Such predictive tools could enable risk adapted screening protocols. For example, a patient with low AI-estimated progression risk might safely be screened every 18–24 months, while high-risk individuals could be monitored more frequently [31]. Tools like the RetinaRisk calculator, which take individual clinical risk factors into account, have shown that screening frequency can be safely adjusted based on patient risk. In a five-year study, RetinaRisk safely extended screening intervals to two years for low risk patients, reducing unnecessary visits without affecting clinical outcomes [32].

Most current OCT platforms feature integrated AI or machine learning algorithms for retinal layer segmentation and central subfield thickness (CST) measurement. However, newer deep learning approaches have demonstrated superior robustness and reproducibility compared with these built-in tools. Goldbach et al. (2025) validated a deep-learning model that automatically measured intraretinal and subretinal fluid in almost 7000 OCT scans, matching the accuracy of human graders [33]. For instance, DL models can delineate intraretinal fluid (IRF) and subretinal fluid (SRF) on serial OCT scans, calculate fluid volume, and detect recurrence earlier than manual reviewers [34,35,36]. This enables clinicians to make data-driven retreatment decisions, especially in busy clinics where subtle changes may be missed.

AI has also been used to detect emerging OCT biomarkers with prognostic significance. These include disorganization of the inner retinal layers (DRIL), hyperreflective foci, and ellipsoid zone disruption, all associated with visual outcomes in DME. In one study, a DL model achieved 88% accuracy in detecting DRIL from OCT scans [37], while another system quantified photoreceptor integrity loss to assist in outcome prediction [38]. Such tools pave the way for staging DME beyond CST, incorporating fine structural biomarkers that are currently underutilized due to grading complexity.

Over time, AI has the potential to identify predictive patterns of disease progression through so-called *digital biomarkers*, subtle imaging features that are *undetectable* to human graders but quantifiable by machine learning. In DR, these may include microaneurysm turnover, vascular remodeling, or changes in lesion dynamics. In DME, metrics such as cumulative fluid index or retinal layer disruption could act as early indicators of disease reactivation. Arcadu et al. demonstrated that AI-derived lesion counts correlated with subsequent development of proliferative DR, highlighting the prognostic value of these data-driven biomarkers [28]. Recent mechanistic studies suggest that cholesterol crystal formation may be a unifying pathogenic driver of diabetic retinopathy, linking metabolic dysfunction to vascular damage and inflammation [39].

AI-based disease monitoring offers frequent, precise measurements of structural changes and enables earlier intervention, supporting a more individualized approach to patient care.

### 3.3. AI in Therapy Monitoring and Treatment Response

Managing diabetic retinopathy (DR) and diabetic macular edema (DME) requires personalized, long-term treatment strategies guided by careful monitoring of therapeutic response. This is especially important in DME, where first-line therapy consists of intravitreal anti-VEGF injections administered over extended periods. Treatment outcomes vary considerably: some patients experience rapid resolution of retinal fluid, while others continue to show persistent or recurrent edema despite regular injections. Artificial intelligence (AI) offers promising tools to refine these decisions by enabling earlier prediction of treatment response, objective monitoring, and proactive retreatment planning [40].

Recent studies have explored whether baseline OCT images, with or without accompanying clinical data, can predict how patients with diabetic macular edema (DME) will respond to anti-VEGF therapy. Cao et al. developed a deep-learning model that extracts structural OCT features, such as fluid volume and retinal layer integrity, and analyzes them using a machine learning classifier. Their random forest model achieved 90.0% sensitivity and 85.1% specificity (AUC 0.923) in identifying good responders, defined as patients who showed both anatomical and functional improvement after three anti-VEGF injections. This AI approach outperformed expert retina specialists in prognostic accuracy [41]. Similarly, Alryalat et al. combined a U-Net convolutional neural network with an EfficientNet classifier, achieving 75% accuracy in differentiating responders from non-responders [42]. By flagging likely non-responders early, such models could help reduce the trial-and-error phase of DME management, for instance, prompting earlier consideration of corticosteroid or focal laser therapy when anti-VEGF treatment proves ineffective.

Beyond baseline prognostication, AI is also being applied to analyze follow-up OCT scans and quantify fluid dynamics during the course of treatment. Rasti et al. demonstrated that volumetric segmentation algorithms can reliably measure fluid volume changes between visits, providing clinicians with consistent metrics to guide retreatment decisions [40].

Recent work has shown that machine learning models combining OCT derived metrics with clinical parameters can effectively predict treatment outcomes in diabetic macular edema (DME). Liu et al. reported that an ensemble model accurately predicted post-treatment central foveal thickness (AUC 0.94) and best-corrected visual acuity (BCVA) (AUC 0.81) after three anti-VEGF injections [43]. Meng et al. applied an OCT-radiomics approach, which extracts quantitative imaging features from OCT scans for machine-learning analysis, to identify patients at risk of persistent DME after treatment (AUC 0.88) [44]. Together, these findings highlight the potential of AI to support individualized treatment planning and optimize injection intervals in DME management.

Innovative AI approaches also include the use of generative adversarial networks (GANs) to simulate post-treatment OCT appearances. Xu et al. developed a GAN that predicted central retinal thickness (CRT) after therapy with an average deviation of 25 microns compared to actual outcomes [45]. Although not yet ready for clinical use, this method illustrates a future in which AI could provide virtual treatment simulations to support therapeutic decision making.

FDA approved AI systems are now beginning to influence clinical practice. The ZEISS PathFinder AI received regulatory clearance in 2022 as a decision support tool for retinal specialists. This AI system analyzes OCT images to detect and quantify retinal fluid in neovascular age-related macular degeneration and DME. Unlike autonomous screening tools, PathFinder provides detailed segmentation and fluid metrics, assisting ophthalmologists in treatment planning and follow-up management [46]. Its approval signals growing regulatory acceptance of advanced AI systems for direct clinical application in retinal disease management.

Although most AI applications to date have focused on DME, emerging tools are being developed for proliferative DR (PDR) management as well. For example, Moosavi et al. applied automated analysis to fluorescein angiography images, quantifying ischemic index and vessel tortuosity to predict the recurrence of DME after initial therapeutic response [47]. This example highlights the potential of multimodal AI models that integrate fundus photography, OCT, angiography, and systemic biomarkers (e.g., HbA1c, renal function) to provide comprehensive treatment guidance.

AI is also transforming how patients are monitored over time. The Notal Home OCT device, first validated in neovascular age-related macular degeneration (AMD), enables patients to perform self-scans from home on a daily basis. Its integrated AI algorithm automatically detects fluid recurrence and alerts physicians, facilitating timely intervention. Clinical studies have shown that this home monitoring system detects fluid in approximately 83% of positive cases, with strong concordance to in clinic OCT assessments [46,48,49]. Building on these results from AMD, similar AI-assisted home monitoring systems are now being investigated for diabetic macular edema (DME). Such tools could support proactive, patient centered management by allowing for early detection of DME recurrence, minimizing unnecessary clinic visits, and ultimately preserving vision while reducing healthcare burden. A prospective observational study further demonstrated that older adults with wet AMD and DME were both willing and able to perform daily self-testing using mobile devices, showing that home based monitoring for diabetic macular disease is both practical and acceptable for patients [50]. Taken together, these innovations highlight the growing role of AI in the management of diabetic macular edema (DME) and diabetic retinopathy (DR). AI applications now make it possible to identify responders early, measure treatment effects, anticipate disease recurrence, and enable remote monitoring. By combining quantitative imaging insights with systemic patient data, AI has the potential to personalize therapy, prevent overtreatment, and make retinal care more efficient.

### 3.4. AI as a Decision Support Tool in Clinical Practice

The true value of artificial intelligence (AI) in diabetic retinopathy (DR) and diabetic macular edema (DME) lies not in replacing clinicians but in enhancing their decision making through seamless integration into routine practice. Rather than acting autonomously, AI systems are designed to analyze imaging and clinical data, providing evidence based insights that support ophthalmologists in diagnosis, disease staging, and treatment planning.

AI-driven screening platforms have already proven valuable for identifying diabetic patients with referable or vision threatening disease who require specialist evaluation. For example, the Moorfields DeepMind OCT AI system, discussed earlier, exemplifies an in clinic decision support tool. In optometric or emergency settings, it can issue real-time alerts for urgent pathology, such as DME requiring prompt anti-VEGF treatment, helping ensure timely referral and reducing the likelihood of missed diagnoses [7,22].

Within retinal clinics, AI-assisted lesion detection and annotation can sharpen diagnostic precision. Algorithms can highlight subtle features such as microaneurysms or small hemorrhages on fundus images that might otherwise go unnoticed. Automated lesion quantification, including hemorrhage counts or ETDRS severity level estimation, has been shown to improve grading consistency [50] and offers objective metrics for tracking disease progression [39]. Similarly, AI applied to OCT can delineate fluid compartments, quantify volumetric changes since the previous visit, and detect emerging biomarkers such as disorganization of the retinal inner layers (DRIL), all of which help clinicians assess disease activity more accurately.

A promising next step is the development of AI systems capable of integrating information across multiple imaging modalities and clinical parameters. In everyday practice, retina specialists combine information from color fundus photography, OCT, fluorescein angiography, and systemic data to guide their decisions. AI could learn to mirror this integrative reasoning and apply it consistently across large patient populations. Early multimodal AI models, such as those combining ultra-widefield fundus imaging with OCT angiography, have shown only modest gains over single modality approaches for DR detection [51]. However, models incorporating systemic parameters such as HbA1c or renal function alongside imaging data have demonstrated improved accuracy in predicting disease progression [29,52].

For AI to have a lasting impact on clinical decision making, it must be integrated into existing workflows. Outputs should be generated almost instantly during patient visits, with interfaces that are intuitive and minimally disruptive. Leading OCT manufacturers are already embedding deep-learning capabilities into their platforms, offering automated layer segmentation, fluid detection, and anomaly alerts. In many cases, clinicians may already be benefiting from these AI-driven enhancements without explicitly realizing it.

Although most FDA clearances to date have focused on autonomous AI for screening, such as IDx-DR and EyeArt, the field is gradually shifting toward decision-support systems that assist with treatment planning. The FDA approved Notal Home OCT device, for instance, enables remote monitoring of age-related macular degeneration (AMD) by using AI to detect fluid recurrence, setting an important precedent for similar DME applications. In 2022, ZEISS Retina AI received approval for detecting retinopathy of prematurity, reflecting growing regulatory confidence in ophthalmic AI. Most recently, ZEISS PathFinder AI was cleared to quantify intraretinal and subretinal fluid in patients with AMD or DME, marking a significant step toward AI-assisted treatment guidance. Unlike autonomous systems that generate simple binary outputs, PathFinder provides detailed, quantitative data that can directly inform injection decisions and follow-up intervals.

Despite these advances, several challenges remain. Clinicians often cite medicolegal liability and the “black-box” nature of AI algorithms as barriers to adoption [53,54]. These concerns are gradually being addressed by framing AI as an assistive tool rather than a replacement, with the final clinical decision always resting with the physician. Evidence suggests that AI support can modestly improve diagnostic accuracy, particularly among non-specialists, by flagging subtle abnormalities that might otherwise go unnoticed [9]. AI can serve as an additional layer of safety by flagging findings that might otherwise be missed, while clinicians provide the essential oversight that keeps interpretation accurate and trustworthy. As AI tools become more explainable and familiar, their acceptance in daily practice is likely to increase.

## 4. Discussion

The advances summarized in this review demonstrate that artificial intelligence (AI) has made significant progress in the detection, staging, and management of diabetic retinopathy (DR) and diabetic macular edema (DME). Deep learning algorithms now match or exceed expert performance in identifying referable DR, quantifying fluid on OCT, and even predicting treatment outcomes. However, the path from laboratory innovation to widespread clinical adoption remains complex and depends on addressing several key challenges.

A major issue is the variability in AI performance across populations, devices, and imaging modalities. Retinal images differ by camera type, image resolution, fundus pigmentation, and lesion presentation, and algorithms trained on homogeneous datasets may underperform in more diverse clinical settings [24]. To mitigate this, large-scale, multiethnic datasets and data-augmentation strategies are being pursued. Recently, *foundation models*, very large AI architectures pre-trained on millions of retinal images, have been introduced, such as RETFound. These models can be fine-tuned for specific tasks and have shown improved generalizability across diseases and populations [55].

Regulatory hurdles also remain significant. AI models must undergo rigorous validation before clinical use, and agencies such as the U.S. FDA require prospective evidence of safety, efficacy, and consistency. As algorithms evolve with continuous data input, new regulatory frameworks for monitoring “adaptive” systems are being developed. The FDA’s distinction between “locked” and “continuously learning” models has provided a foundation for conditional approvals based on ongoing performance evaluation [56]. In parallel, large-scale, prospective clinical trials are needed to confirm that AI implementation not only matches clinician performance but also improves patient outcomes and cost-effectiveness.

There are also practical challenges in implementation. Some clinics lack the IT infrastructure or internet connectivity required to support AI platforms, and staff may need additional training to interpret AI outputs reliably [57]. Financial barriers persist as well: although Current Procedural Terminology (CPT) reimbursement codes have been introduced for autonomous AI screening in the U.S., current payments may not fully offset the costs of deployment and maintenance [58,59]. For broader adoption, AI systems must demonstrate both clinical and economic value, such as reducing unnecessary referrals, optimizing injection timing, or streamlining workflows. Successful pilot programs that clearly improve efficiency and patient care will be key drivers of clinical integration.

Ethical and legal considerations are equally important. Data privacy and patient consent remain central concerns, particularly given the scale of data required for algorithm development. Bias mitigation is another critical challenge: algorithms must perform equitably across diverse populations to avoid perpetuating health disparities [60,61]. Transparency and interpretability are also essential for clinician trust. Advances in explainable AI aim to produce human-interpretable outputs, for example, highlighting specific retinal features that influenced a recommendation, thereby increasing confidence and accountability [53,62]. It should also be noted that this review did not include a formal risk-of-bias or study-quality assessment, given the considerable heterogeneity among included studies in design, dataset composition, and outcome reporting.

Looking ahead, AI in ophthalmology is moving toward more holistic and personalized applications. Multi-task models that can diagnose, grade, and predict disease progression from a single image may soon replace the task-specific tools used today. Predictive analytics that combine longitudinal imaging with systemic data could help identify disease activity earlier and guide truly individualized follow-up plans.

Another promising direction is federated learning, which enables AI models to be trained collaboratively across multiple institutions without exchanging patient data, enhancing both generalizability and data privacy.

In DME specifically, AI may soon guide precision therapy by linking OCT scans with optimal treatment strategies. Home-based monitoring, such as the Notal Home OCT platform with integrated AI analysis, illustrates how remote disease management and patient engagement could be transformed. Future iterations of smartphone-based retinal imaging, coupled with AI, may further democratize access to care, especially in underserved regions.

In addition to imaging-based applications, recent work has explored no-code, large language model (LLM) approaches for DR and DME risk prediction using tabular clinical and laboratory data. Choi et al. demonstrated that ChatGPT-4 can develop validated logistic regression models without manual coding, achieving AUC values of 0.786 for DR and 0.835 for DME, and generating a web-based risk calculator deployable in primary care settings without retinal imaging. This emerging direction highlights how LLMs may lower technical and infrastructural barriers for early risk stratification and screening in underserved areas [63].

Finally, this review provides a qualitative synthesis of current AI applications in DR and DME rather than a quantitative meta-analysis. A narrative approach was chosen due to the methodological and clinical heterogeneity of existing studies, which precluded formal data pooling. Future systematic reviews with broader database coverage (including Embase, IEEE Xplore, and Cochrane Library) and formal bias assessment will be valuable as the field matures. This narrative review did not include all potentially relevant databases, did not apply dual-independent screening, and did not perform a formal risk-of-bias assessment. These methodological constraints may introduce selection and publication bias and should be considered when interpreting the findings.

## 5. Conclusions

In the decade since deep learning entered ophthalmology, artificial intelligence has progressed from experimental promise to practical application in diabetic eye care. For retina specialists, AI now provides tangible benefits: expert-level detection of diabetic retinopathy (DR) and diabetic macular edema (DME), consistent disease staging, quantitative OCT analysis, and personalized treatment support. Clinical studies have shown that AI can enhance diagnostic accuracy, expand screening coverage, and help prioritize urgent cases, translating technical performance into meaningful patient outcomes [7,20,59].

Still, full integration of AI into ophthalmic practice will require overcoming barriers related to clinical validation, infrastructure, reimbursement, ethics, and clinician trust. Encouragingly, new developments, such as foundation models, explainable AI, and multimodal decision-support systems, are beginning to address these challenges.

If implemented thoughtfully, AI will not replace the retina specialist but will serve as a valuable partner, enabling more efficient, equitable, and proactive diabetic eye care. Looking ahead, we envision a future in which routine DR and DME screening is largely automated, specialists can focus on treatment and complex decision-making, and each management plan is guided by data-driven insights. Ultimately, these advances should help preserve vision and improve quality of life for millions of people living with diabetes worldwide.

## Data Availability

No new data were created or analyzed in this study.
